# 3D Printed Conformal Strain and Humidity Sensors for Human Motion Prediction and Health Monitoring via Machine Learning

**DOI:** 10.1002/advs.202304132

**Published:** 2023-11-08

**Authors:** Yanbei Hou, Ming Gao, Jingwen Gao, Lihua Zhao, Edwin Hang Tong Teo, Dong Wang, H. Jerry Qi, Kun Zhou

**Affiliations:** ^1^ HP‐NTU Digital Manufacturing Corporate Lab School of Mechanical and Aerospace Engineering Nanyang Technological University Singapore 639798 Singapore; ^2^ Singapore Centre for 3D Printing School of Mechanical and Aerospace Engineering Nanyang Technological University Singapore 639798 Singapore; ^3^ 3D Lab HP Labs HP Inc. Palo Alto CA 94304 USA; ^4^ School of Electrical and Electronic Engineering Nanyang Technological University Singapore 639798 Singapore; ^5^ School of Mechanical Engineering Shanghai Jiao Tong University Shanghai 200240 China; ^6^ The George Woodruff School of Mechanical Engineering Georgia Institute of Technology Atlanta GA 30332 USA

**Keywords:** 3D printing, humidity sensors, machine learning, strain sensors

## Abstract

Wearable sensors have garnered considerable attention due to their flexibility and lightweight characteristics in the realm of healthcare applications. However, developing robust wearable sensors with facile fabrication and good conformity remains a challenge. In this study, a conductive graphene nanoplate‐carbon nanotube (GC) ink is synthesized for multi jet fusion (MJF) printing. The layer‐by‐layer fabrication process of MJF not only improves the mechanical and flame‐retardant properties of the printed GC sensor but also bolsters its robustness and sensitivity. The direction of sensor bending significantly impacts the relative resistance changes, allowing for precise investigations of joint motions in the human body, such as those of the fingers, wrists, elbows, necks, and knees. Furthermore, the data of resistance changes collected by the GC sensor are utilized to train a support vector machine with a 95.83% accuracy rate for predicting human motions. Due to its stable humidity sensitivity, the sensor also demonstrates excellent performance in monitoring human breath and predicting breath modes (normal, fast, and deep breath), thereby expanding its potential applications in healthcare. This work opens up new avenues for using MJF‐printed wearable sensors for a variety of healthcare applications.

## Graphical abstract

A successful formulation of conductive ink is achieved for advanced multi jet fusion printing. Through precise control of the ink type and distribution, durable and flame‐retardant multi‐layered sensors with remarkable sensitivity to strain and humidity are produced. The conformal sensors have potential applications in human motion prediction and health monitoring. Leveraging machine learning techniques, the prediction accuracy exceeds 95%.

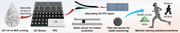

## 1 Introduction

Wearable sensors have gained extensive attention in the past decade in diverse fields ranging from electronic skins, intelligent human/machine interactions, soft robotics, and energy harvesting.^[^ ^1^ ^]^ There is a growing interest in developing wearable sensors for regular and continuous monitoring of human motions and health conditions.^[^ ^2^ ^]^ With the progress of information technology in recent years, data‐driven healthcare monitoring based on distributed sensors can provide real‐time safety‐related information.^[^ ^3^ ^]^ Monitoring such information is essential for prompt medical treatment when an individual's health indicators are abnormal and to avoid missing the optimal treatment time. Traditional fabrication methods are mainly coating, casting, vacuum deposition, and polymerization,^[^ ^4^ ^]^ and the sensors are then tailored and assembled to fit the movements of humans. Limited by fabrication techniques, traditional sensors have simple structures, complicated fabrication processes, and poor conformity with the human body (Table S1, Supporting Information).^[^ ^5^ ^]^ Self‐powered wearable sensors with sustainable and independent operation are developed, which can be driven by mechanical, thermal, solar, biochemical, or radio frequency (RF) energy harvesting.^[^ ^6^ ^]^ To enhance conformality and function integration, conformal manufacturing techniques, such as transfer printing and direct printing, are being investigated for fabricating and integrating deformable sensors onto 3D freeform surfaces.^[^ ^7^ ^]^ It is of great interest to explore further possibilities for scalable fabrication methods that enable the fast production of sensors with multifunctionality and the direct fabrication of devices with integrated sensors.

The special multilayered structures of peacock feathers, butterfly wings, and natural shells endow them with superior optical and mechanical properties.^[^ ^8^ ^]^ Following the examples found in nature, it is evident that special properties can be obtained by using multilayer‐structured composites.^[^ ^9^ ^]^ For example, bio‐inspired composites designed for natural shells can achieve notable stiffness and toughness.^[^ ^10^ ^]^ Multilayered polymer composites with responsiveness and functionality could be engineered through appropriate material selection. It has been increasingly recognized that the assembly method has a substantial impact on the physicochemical properties of multilayered polymer composites.^[^ ^11^ ^]^ However, the methods for preparing multilayer structures are complicated, such as spin coating, spraying, and deposition.^[^ ^12^ ^]^ None of these methods can directly fabricate multilayered products with complex structures. Therefore, a new preparation method should be adopted to prepare such multilayer structures with excellent performance.

3D printing of custom functional structures with controllable geometry and design can address the limitations of traditional manufacturing methods by providing the necessary conformality.^[^ ^13^ ^]^ The challenge of efficiently producing wearable devices using 3D printing technology persists due to limitations in the speed of production. Powder bed fusion (PBF) is an important category of 3D printing technology as defined by ISO/ASTM52900‐21. It involves layer‐by‐layer selective fusion of powder materials to form 3D objects by an energy source.^[^ ^14^ ^]^ Multi jet fusion (MJF) is a new PBF technique that utilizes multiple jets to selectively fuse powdered polymer materials into high‐resolution parts with support‐free structures, offering a more rapid fabrication approach compared to other off‐the‐shelf 3D printing technologies. In MJF, two types of printing inks (i.e., the fusing agent (FA) and the detailing agent) are selectively deposited on each powder layer to form the desired pattern according to the computer‐aided design model before the powder layer is irradiated by infrared lamps. The black FA absorbs heat energy from the infrared lamps and enables the powder particles in selective regions to fuse together. The colorless detailing agent is deposited at the boundaries of the fusing regions to lower the temperature of the surrounding powder particles, thus ensuring intricate details of the printed parts. MJF allows for the selective deposition of multiple inks to print multifunctional integrated sensors, as demonstrated in our earlier work where we established that a self‐prepared functional ink can significantly improve the electrical conductivity and mechanical properties of the printed components.^[^ ^15^ ^]^ However, the advantages of the layer‐by‐layer processing technology have not been thoroughly investigated.

Conductive materials such as graphene nanoplates (GNPs), carbon nanotubes (CNTs), and some metals are often combined with flexible substrates to fabricate sensors.^[^ ^16^ ^]^ Assembly of GNPs with CNTs can yield hybrid materials with new structural characteristics and properties different from those of the individual components,^[^ ^17^ ^]^ which can lead to better functional performance. Combining GNPs and CNTs to design laminated composites has been extensively studied for applications in supercapacitors, transparent conductors, and lithium‐ion batteries.^[^ ^18^ ^]^ The performance of these materials has been greatly improved because CNTs can bridge the electron transfer defects and effectively prevent the irreversible aggregation of GNPs. Furthermore, the hybrids have unusual intercalation properties, high storage, and chemical stability.^[^ ^19^ ^]^ Therefore, the GNPs–CNTs hybrids are expected to increase the potential of GNPs or CNTs as a free‐standing electrode material.

In this work, we prepared a conductive ink using GNPs and CNTs. The GNPs–CNTs (GC) ink was used as the FA for MJF printing to fabricate multilayered strain and humidity sensors, which can be used to predict human motions and health conditions. The presence of CNTs blocks the aggradation of GNPs, while GNPs promote the formation of conductive paths. With the assistance of computer‐aided design, MJF‐printed parts have great conformity with the shape of human joints. To further explore the sensing performance of the GC sensor, a support vector machine (SVM) algorithm was used to classify raw data of different motions. Statistical characteristics of the collected data were thoroughly investigated. Moreover, the fabricated sensor has a humidity sensitivity, which can be used to detect the vital signs of users.

## 2 Results and Discussion

**Figure 1:**
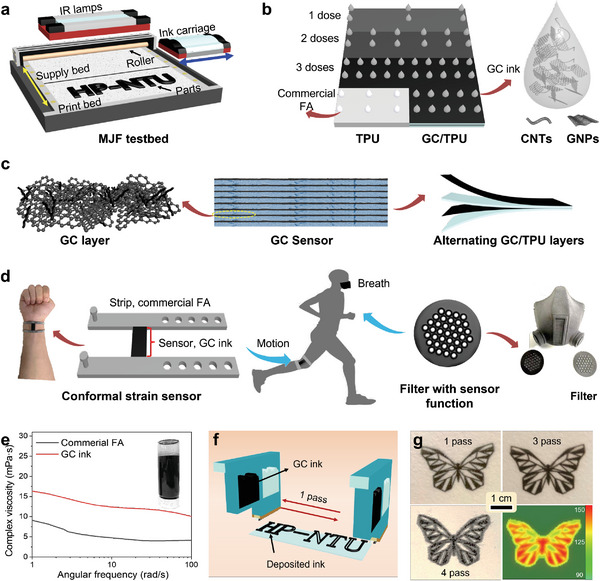
Fabrication and application of GC sensors. a) Schematics of the MJF testbed setup; b) Selective ink jetting during printing and schematic illustration of the GC ink; c) Illustrations of the components of a GC sensor; d) Application scenarios of GC sensors; e) Rheological behaviors of the GC ink; f) The definition of one pass of MJF printing; g) Digital images and IR image of printed butterfly patterns with different passes.

During the MJF printing process, the ink carriage moves side to side above the print bed to deposit inks in the designed pattern on the powder bed (**Figure** **1**a). The MJF printer not only regulates the type and placement of the deposited ink but also adjusts the dose of inkjet based on the sample structure design, resulting in the production of materials with varying conductive properties. Figure 1b illustrates a layer printed with a specific technique for depositing ink in advanced MJF printing, where the inkjet dosage and type of ink can be flexibly adjusted during the ink carriage movement. The inks used for MJF printing in this work include the commercial FA and the self‐prepared GC ink. Thermoplastic polyurethane (TPU) powders are used to produce adaptable and flexible polymer layers that are capable of conforming to the shape deformation of the human body. The advanced MJF printing process of a multilayered sensor can be divided into six steps, as shown in Figure S1 (Supporting Information). The layer‐by‐layer fabrication process of a sensor (Figure 1c) not only reduced the consumption of conductive fillers but also bolstered the mechanical strength of the printed GC sensor. The MJF‐printed sensors of various shapes possess exceptional conformity and can be employed for motion prediction and health monitoring purposes (Figure 1d).

### 2.1 Characterization of the GC Ink

Exfoliated GNPs and well‐dispersed CNTs were obtained after probe sonication treatment, confirmed by scanning electron microscopy (SEM) (Figure S2a, Supporting Information) and transmission electron microscopy (TEM) (Figure S2b, Supporting Information), respectively. The dispersion of GNPs and CNTs in the GC ink was shown in Figure S2c (Supporting Information), whereby CNTs attached to the surface of GNPs and prevented the agglomeration of GNPs. The resulting mixture had low viscosity and a solid content of 3.12 mg ML^−1^, in which the weight ratio of polyvinylpyrrolidone (PVP), GNPs, and CNTs is 2:1:1. A self‐supporting GC film (≈300 µm in thickness) with good flexibility and conductivity can be obtained by drying a certain amount of the GC ink at 80 °C in an oven. Figure S2d (Supporting Information) displays a fractured surface of the GC film, revealing distinct features of CNTs and GNPs. The GC film demonstrated high thermal sensitivity and was capable of deformation when exposed to an external heat source (Figure S3, Supporting Information).

The GC ink showed excellent stability as it did not settle to the bottom of the bottle after 2 months (Figure 1e, inset). In contrast, the mixture containing only GNPs tended to form a precipitate at the bottom of the bottle, thus proving that the presence of CNTs prevented the aggregation of GNPs. The inverse Ohnesorge number *Z* indicates the printability of an ink. When *Z* falls within the range of 1–10, the ink droplet jetting behaviors are well controlled, indicating that the droplet formation and ejection are stable.^[^ ^20^ ^]^ The value of *Z* can be calculated by

(1) $$Z=\sqrt{\rho \gamma d}/\eta$$

where *ρ*, *γ*, and *η* denote the density, surface tension, and complex viscosity (at 100 s^−1^) of the ink, respectively, while *d* denotes the diameter of the nozzle (16 µm). The density values of the commercial FA and the self‐prepared GC ink were 1.105 and 1.060 g cm^−3^, respectively. The surface tension values of the commercial FA and the GC ink were 31.6 and 57.4 mN m^−1^, respectively, measured by a contact angle meter. Based on the above parameters, the *Z* values of the commercial FA and GC ink were calculated to be 5.565 and 3.173, respectively, within the range of stable ink droplet jetting.

The resolution of the conductive features formed by the GC ink was also evaluated. The MJF printing conditions are highly flexible, allowing for the adjustment of ink‐dispensing volume by altering the ink‐dispensing times. This enables precise control over the conductivity of printed parts in specific regions. A reciprocating motion of the ink carriage (equivalent to two ink‐dispensing times) is defined as 1 pass, as depicted in Figure 1f. The printing resolution of the GC ink was proved by patterning a complex butterfly shape on TPU powder (Figure 1g). The GC ink demonstrated excellent precision and versatility in printing complex parts, as evidenced by the ability to produce lines thinner than 200 µm. With an increased number of passes (four in this case), the pattern exhibited a sudden decay in resolution, probably due to diffusion of the excess ink and slow drying. This could be detrimental to the dimensional control of the final component. Therefore, the maximum number of passes used in this work is three.

The photothermal property of the GC ink affects the fusion of polymer particles. To investigate the influence of the ink‐dispensing volume on the photothermal performance of the printed parts, a simulated solar irradiation setup was assembled, as shown in Figure S4a (Supporting Information). All printed samples attained a comparable stable temperature range (115–120 °C) a few minutes after lamp activation under 1 kW m^−2^, irrespective of the number of passes utilized, suggesting that the amount of ink dispensed has minimal impact on the photothermal performance (Figure S4b, Supporting Information). The rapid heating of the powder implies good heat absorption capabilities of GC ink. This feature is advantageous in melting the polymer powders and generating high‐performance MJF printed components.

The conductivity of the ink affects the electromechanical performance of the printed components, which was tested by a four‐probe conductivity meter and a source meter. The overlapped fillers form conductive paths and endow the self‐supporting GC film with a low resistance, the electrical conductivity of which was 3.18 S m^−1^. To test the influence of the ink‐dispensing volume on the electrical conductivity, multilayered GC/TPU parts with the same size (10 mm × 20 mm × 1.2 mm) were printed, and each part consists of 20 layers of TPU and GC. The ink‐dispensing volume was found to have a significant impact on the conductivity of printed parts, with conductivity increasing as the number of passes increased. The conductivity increased from 1.23 × 10^−5^ S m^−1^ (1 pass) to 4.17 × 10^−3^ S m^−1^ (2 passes) and 1.48 × 10^−2^ S m^−1^ (3 passes) along the 20 mm direction, indicating that the GC ink is suitable for producing low‐voltage sensors. Meanwhile, the TPU's resistance exceeded the capacity of the tester (5 × 10^−5^ S m^−1^), even after being subjected to three passes of commercial FA deposition, which suggests that it has a low level of conductivity. The detailed data can be found in Table S2 (Supporting Information). The volt‐ampere characteristic curve shows that the linear fitting lines precisely match the collected data points (Figure S5a, Supporting Information). This indicates that the resistance of printed GC/TPU composites remains stable over the entire voltage range tested, which spans from 0 to 100 V.

The printability and conductivity of the GNPs and CNTs solutions were also evaluated and compared to those of the GC ink. The printability of the GNP solution is hindered by its poor dispersion, while the utilization of the CNTs solution for printing CNTs/TPU composites has resulted in insufficient conductivity (as illustrated in Figure S5b, Supporting Information). The high resistance of the CNTs/TPU composite can be attributed to the acidification process used to enhance the dispersion of CNTs in water, which unfortunately reduces its electrical conductivity. The dried inks that incorporate GNPs exhibit favorable conductivity (>0.59 S m^−1^). However, when the GNPs content exceeds 50 wt.%, the material tends to precipitate and cause clogging in the nozzle, thus hindering the smooth printing process of MJF. The ink with 50 wt.% GNPs shows the best conductivity in the printable inks. Hence, the GC ink, comprising equal amounts of GNPs and CNTs, is a well‐suited functional additive for MJF printing of sensors due to its favorable stability, printability, and conductivity properties.

### 2.2 Characterization of Printed GC/TPU Composites

**Figure 2:**
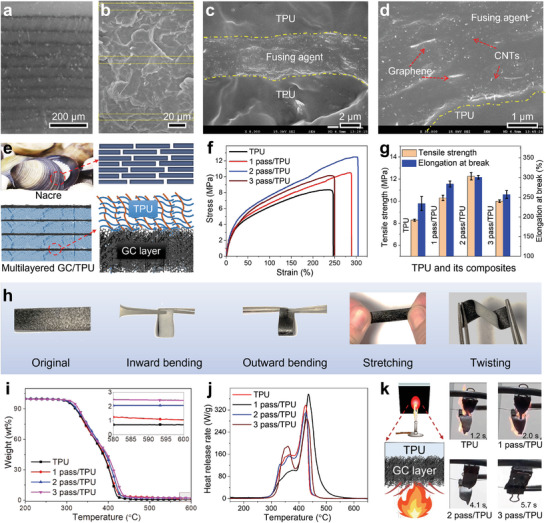
Characterization of GC/TPU composites. a) Optical microscope image of the fractured surface of 3 pass/TPU sample; b–d) SEM images of 3 pass/TPU sample with different magnifications; e) Illustrations of the nacre‐inspired structure of the printed GC/TPU composites; f) Stress–strain curves and g) collected data from tensile testing of TPU and GC/TPU composites; h) Digital images of the GC/TPU sample with different deformations; i) TGA and j) HRR curves of TPU and GC/TPU composites; k) Combustion behaviors of printed GC/TPU composites.

In the multilayered GC/TPU composites, the polymer layer and the conductive layer were stacked alternately. The fractured section of the MJF printed GC/TPU composites under different magnifications. The TPU layers (grey) and GC layers (black) were clearly observed by an optical microscope, as shown in **Figure** **2**a. The interface between the TPU and GC layer was blurred, and no cracks were observed by SEM in all views, indicating good compatibility between the two layers (Figure 2b–d). Compared to conventional methods of preparing conductive polymer composites, this MJF printing approach has several advantages. First, it reduces the usage of conductive fillers and enables the fabrication of parts with complex structures. Secondly, it maximizes the electric robustness of the sensor, as each unit can function independently.

In the GC/TPU composite, which consists of alternating TPU layers and GC layers formed by three ink‐dispensing passes (referred to as 3‐pass/TPU), the thickness ratio between the TPU layer (with an average thickness of 63.215 µm) and the GC layer (with an average thickness of 3.359 µm) is ≈18.82 (Figure 2c). Therefore, the volume percentage of the GC layer in the GC/TPU composite can be calculated as 5.05 vol.%. In the magnified image of the thin layer (Figure 2d), features of layered GNPs and linear CNTs can be clearly observed, confirming that the layer was formed by GC ink. The weight percentage of the conductive layer can be calculated by

(2) $$\frac{\rho_{F}V_{F}}{\rho_{P}V_{P}+\rho_{F}V_{F}}\times 100\%=C$$

where ρ_F_ and ρ_P_ denote the density of the filler (1.528 g cm^−3^) and polymer (1.183 g cm^−3^), while *V*_F_ and *V*_P_ denote the volume of the filler and polymer. Therefore, the weight percentage of the conductive layer, *C*, was 6.42 wt.%. Considering the weight percentage of conductive fillers in the GC layer was 43.00 wt.% (calculated from TGA data in Figure S6, Supporting Information), the overall concentration of the conductive fillers in the 3‐pass/TPU was 2.76 wt.%.

A robust conductive layer is important for ensuring the stable performance of sensors. The multilayered structure of the GC/TPU composite resembles the hierarchical multilayer structure found in shells (Figure 2e). Multilayered interfaces can regulate stress distribution, stress transfer, and propagation of micro‐cracks.^[^ ^21^ ^]^ The mechanical performance of the self‐standing GC film and fabricated samples was tested by a tensile machine (3360 Series Dual Column Table Frames, Instron, USA). The GC film exhibited a high tensile strength of 45 MPa, but its elongation at break was only approximately 15% (Figure S7, Supporting Information). Figure 2f,g shows the mechanical performance of the printed samples with different inkjet passes. The tensile strength and elongation at break of the GC/TPU composites were obviously increased, compared to that of neat TPU. Without sacrificing the elongation at break, the tensile strength of the GC sensor increased by 25.62%, 49.55%, and 21.37% when the number of printing passes was 1, 2, and, 3, respectively. Although the mechanical performance of the GC/TPU samples was impaired by the increasing number of inkjets, it was still better than that of neat TPU. The rigid and strong GC film works as the reinforcement layer, which confronts the external force and makes the composites strong.^[^ ^22^ ^]^ The rigidity of the GC layer weakens the elongation at the break of the printed TPU composite. However, the printed part retains the flexibility of TPU and can be bent inward/outward, stretched, and twisted, as shown in Figure 2h. The flexibility of the printed GC/TPU composites ensures that they can be used as wearable devices in the healthcare field.

Evaluation of the thermal stability of the MJF‐printed GC/TPU composites can help determine its application scenarios. The combination of the GC layer and TPU matrix was beneficial in improving the initial degradation temperature (temperature at 5 wt.% weight loss) of the printed sensors (Figure 2i). The increased amount of char residues also confirms that the thermostability of the TPU composites has been improved. Given the thermal weight loss behavior of GC, the mass fraction of the combustion residue in the GC/TPU composite is significantly higher than the amount of GC added. This suggests that GC plays an effective role in fixing the char layer, which is likely due to the barrier effect of the carbon networks formed by CNTs and GNPs.

Peak heat release rate (PHRR) is an important parameter used to evaluate the fire safety performance of polymer composites.^[^ ^23^ ^]^ The PHRR value of pure TPU was 396.2 W g^−1^, and it decreased with the increasing deposition pass of the GC ink (Figure 2j), which can be ascribed to the barrier effect of the thermostable GC layer. The 3 pass/TPU had the lowest PHRR value (287.1 W g^−1^), signifying that the material has the best flame‐retardant properties. A lower PHRR value means that less heat will be released per unit time, which will suppress the development of fires.^[^ ^24^ ^]^ The results demonstrated that the flame retardancy of the printed parts was enhanced by the alternating layers of GC and TPU.

To provide further evidence of the barrier effect of the GC layer, a self‐designed experiment was carried out. The black surface of the printed GC/TPU composites (10 mm × 20 mm × 1.2 mm) was positioned above a 2 cm flame and the time taken for ignition to occur was measured, as illustrated in Figure 2k (left). Compared to that of neat TPU (1.2 s), the ignition time of the 3‐pass/TPU sample was longer (5.7 s). The GC layer only delays ignition but cannot completely prevent the 3‐pass/TPU from burning. However, the burned 3‐pass/TPU composite kept its original shape, while other samples were blown apart, demonstrating the barrier effect of the GC layer (Figure 2k, right). The GC layer, which has high thermal stability, acted as a barrier to heat transfer, and prevented the spread of fire, thereby improving the flame retardancy of the GC/TPU composites. The improved thermal stability and flame retardancy of the multilayered GC/TPU samples can enhance the fire safety performance of such materials in extreme environments, such as those encountered in fire rescue operations.

### 2.3 Strain and Bending Sensing Properties of the GC Sensor

**Figure 3:**
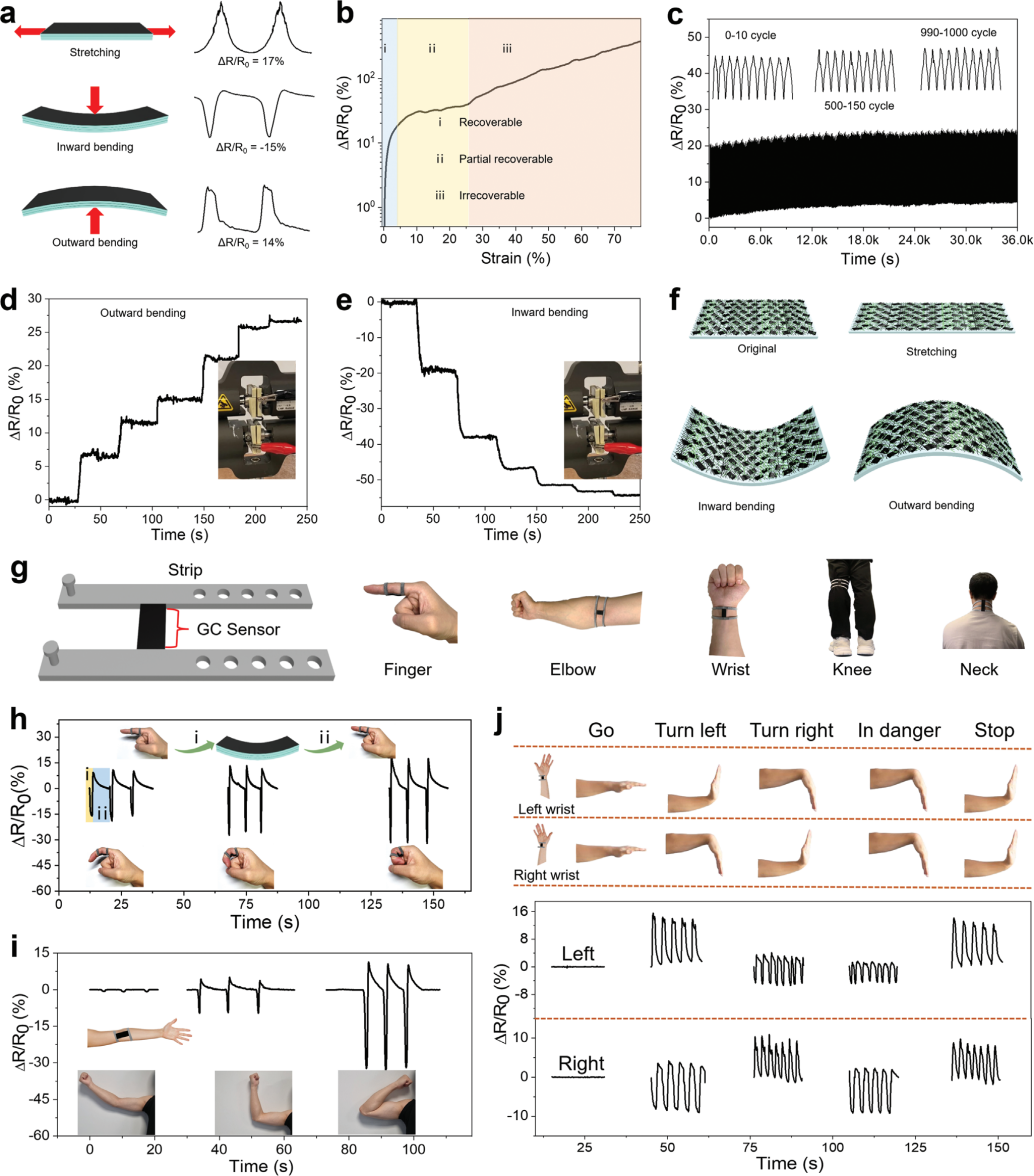
Electromechanical properties of the as‐fabricated GC sensors and human motion monitoring tests. a) Detection signals of the GC sensor under different deformations; b) Change in normalized resistance against strain; c) Cyclic test results of 1000 stretch–release cycles of the GC sensor tensile bar with 5% elongation; The GC sensor monitors the response signals of d) outward bending and e) inward bending; f) Schematic illustration of the changes in the conductive layer under external forces. g) The design and applications of the printed GC sensors; Output signals of the GC sensor collected at the bending of h) finger and i) elbow; j) Discrimination of emergency signs based on two GC sensors worn on both wrists.

The as‐fabricated GC sensor has a strain and bending sensitivity. The shapes and intensities of output signals (the relative resistance change, Δ*R*/*R*_0_) were significantly different during stretching, inward bending, and outward bending (**Figure** **3**a). The resistance of the GC sensor increased with tensile and outward bending but decreased with inward bending. The sensor's shape change can be monitored by analyzing the distinct signals generated due to deformation, and the bending direction changes can be predicted from these signals. These features can be applied to monitor human motions such as complex joint motions of the wrist, neck, knee, and elbow.^[^ ^25^ ^]^

Figure 3b shows the Δ*R*/*R*_0_ of the printed sensor as a function of tensile strain *ε*. The relationship between Δ*R*/*R*_0_ and *ε* exhibited a slow progression in the initial stages, followed by an exponential rise at high *ε* levels. This trend corresponds to the nonlinear region in the piezoresistive response, resulting from an increase in the tunnel distance between the fillers. The gauge factor *G*, defined as the slope of the plot for Δ*R*/*R*_0_ against *ε*, serves as a crucial indicator for assessing the sensitivity of a strain sensor.^[^ ^26^ ^]^ The conductive network composed of GNPs and CNTs can be stretched under external forces, resulting in an increase in resistance and high sensitivity to stretching (*G* = 20.1 at the elongation range of 0–5%). Despite an increase in the stretching ratio of the sensor, the conductive network still remains interconnected, thus causing minimal impact on the overall resistance change and the *G* value (*G* = 2.3 at the elongation range of 6–26%). As the conductive path starts to break with further stretching, the resistance sharply increases, leading to high sensitivity to stretching (*G* = 360.8 at the elongation range of 26–70%). Besides, the large elongation broke the conductive GC layer, and irreversible sensitivity was obtained.^[^ ^27^ ^]^ As shown in Figure 3c, at the same tensile elongation (5%), the change in ΔR/R_0_ value remained stable after 1000 cycles of strain deformation, confirming the stable electromechanical performance of the MJF‐printed multilayer GC strain sensor.

To further investigate its performance in different bending directions, a series of gradient bending was applied to the GC sensor. The value of Δ*R*/*R*_0_ has a strong correlation with the curvature (Figure 3d,e). For outward bending, the value of ΔR/R_0_ increased as the curvature became larger. For inward bending, the resistance changes of the sensor decreased as the curvature increased. Figure 3f schematically illustrates the change in conductive layers during deformations. The outward bending generates a stretch‐like effect on the conductive layer, thus increasing the electrical resistance of the GC sensor gradually when gradient bending is applied.^[^ ^28^ ^]^ Conversely, when bending inward, the conductive material is compressed, making the conductive path denser and thus reducing the resistance. The significant differences in the electrical output signals, which are generated due to the deformation of the sensor, demonstrate excellent stability. This stability allows for estimating the sensor's deformation by analyzing the obtained electrical signals, and such estimation can be utilized for predicting remote actions.

The monitoring of human activities requires wearable sensors attached to different body parts to recognize motion. The GC sensors fabricated using MJF printing exhibit characteristics of flexibility, lightweightness, and the ability to be tailored for optimal conformity with joints. By adjusting the dimensions of the strips and GC sensors (as shown in Figure 3g), the printed strain sensors can effectively accommodate diverse joints in the human body, including fingers, wrists, elbows, necks, and knees. To explore the practical application of the GC sensors, a series of GC sensors with stripes of different lengths were printed and worn on the joints mentioned above.

The GC sensor has an inward bending mode when the joints are bent, and the value of Δ*R*/*R*_0_ gradually increases with the bending of the finger (Figure 3h, process i). The deformation process has minimal effect on the original resistance of the GC sensor. When the finger is straightened and the GC sensor returns to its original shape, the resistance change gradually decreases to zero (process ii). Therefore, the degree of bending of the finger can be assessed according to the peak value of the signal. A similar phenomenon also occurs in the GC sensor worn at the elbow joint. The output Δ*R*/*R*_0_ of the GC sensor when the arm is bending at ≈30°, 90°, and 140° is depicted in Figure 3i. Correspondingly, with the increase in the bending angle, the GC sensor shows an increasing Δ*R*/*R*_0_ value. The performance of fabricated GC bend sensors presents opportunities for monitoring the movement of specific body parts. Additionally, the range of the output signal can be utilized to evaluate the magnitude of motion.

Furthermore, some emergency hand signals, such as “go”, “stop”, “turn right”, “turn left”, and “in danger” can be easily discriminated based on two GC sensors worn on the right and left wrists (Figure 3j). As the wrist orientation changes, the output signal of a wearable device can exhibit three distinct characteristics: stationary, lifted, or pressed. By synchronizing the movements of both wrists, it is possible to generate nine unique combinations, corresponding to nine possible commands. Nevertheless, this study only utilized five of these combinations to prevent potential signal overlap and confusion. Compared to other reported sensors, the MJF‐printed GC sensor cleverly utilizes the signal changes caused by its bending direction to effectively generate the five types of commands.^[^ ^29^ ^]^ In situations where physical movement is limited, a clear message can be sent out through simple wrist movements. As shown in Figure S8 (Supporting Information), the printed sensor can also fit on the neck, and the output signals change significantly as the joints move. The sensor's signal remained unaffected by the high frequency of continuous movements, indicating its ability to operate consistently for a prolonged duration. This GC sensor can be widely and quickly applied for human motion monitoring without a complicated preparation process.

### 2.4 Motion Prediction with Machine Learning

**Figure 4:**
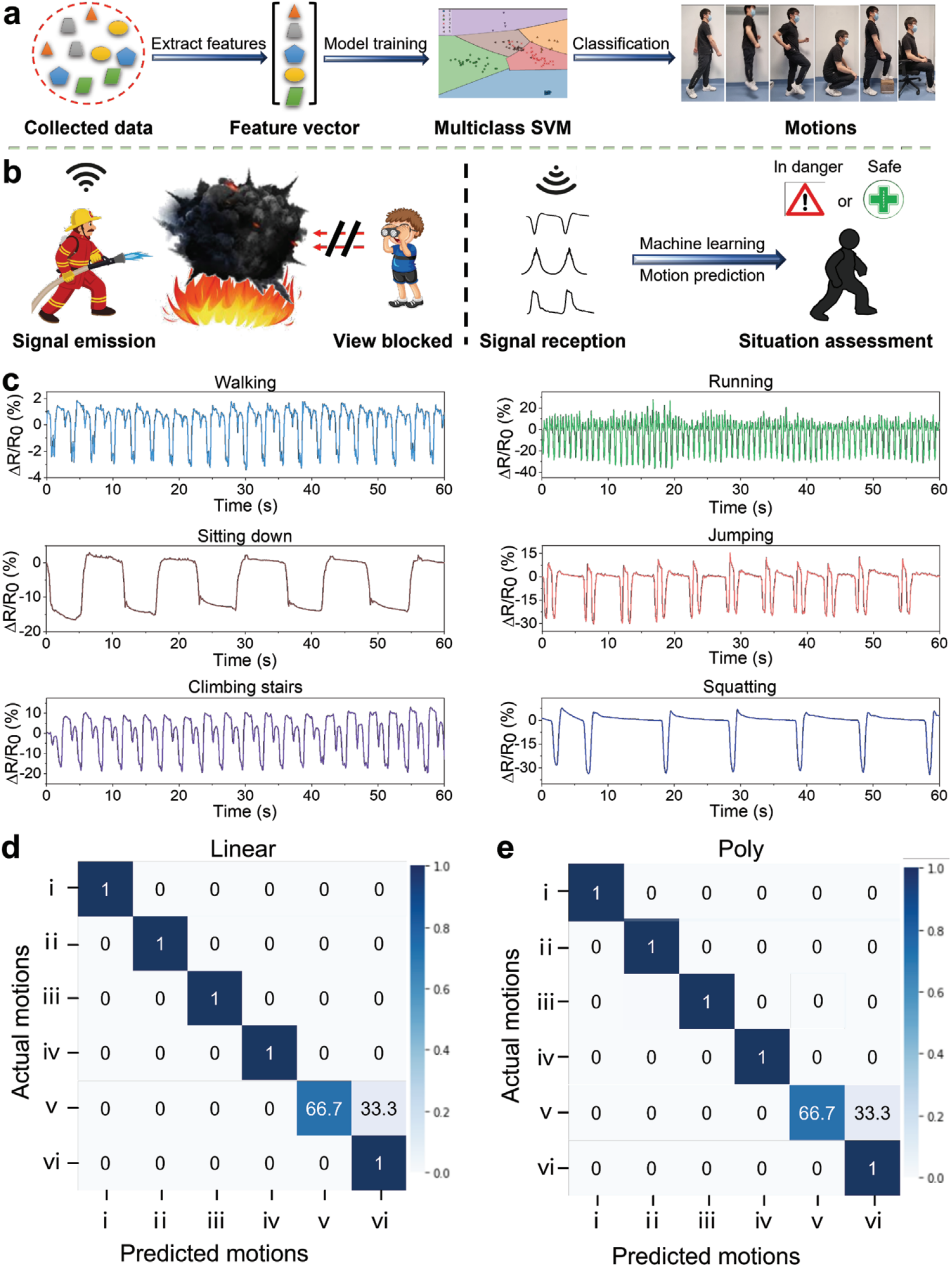
Working mechanisms of ML and its potential applications. a) Schematic of a multiclass SVM classifier for motion prediction; b) The potential application scenario of the fabricated GC sensors; c) Collected data of different motions; Confusion maps for SVM outcome with d) linear and e) polynomial kernel functions, i) walking, ii) running, iii) sitting down, iv) jumping, v) climbing stairs, and vi) squatting.

Interpreting large amounts of sensing data is time‐consuming and labor‐intensive. Machine learning (ML) provides an opportunity to automate this process while achieving equal or higher accuracy than its human counterparts. The ML algorithms can identify useful patterns from training data to make motion predictions, and adaptively improve the prediction accuracy in an iterative learning process. For a motion classification task based on sensing signals, many ML algorithms such as decision trees and k‐nearest neighbors have been utilized.^[^ ^30^ ^]^ Among these algorithms, the SVM stands out due to its high efficiency and simplicity.^[^ ^31^ ^]^ It is a powerful tool for binary classification and can be extended to handle multiclass classification with the use of a one‐versus‐rest approach. Therefore, a multiclass SVM classifier for signal processing was developed. It was trained with sensor data to automatically determine human motions (**Figure** **4**a).

With the help of the SVM model, a large volume of real‐world data can be analyzed simultaneously without the risk of missing or misinterpreting critical signals. This capability is important for the automatic monitoring of human motions in scenarios where visual observation is obstructed due to dense smoke or where infrared thermal imagers are disrupted by heat waves. By collecting signals from the MJF‐printed wearable GC sensors and promptly identifying the type of motion, immediate assistance can be dispatched in times of peril. Therefore, these MJF‐printed wearable GC sensors with improved flame retardancy could be applied for workers on fireground to monitor their motions for personal protection applications (Figure 4b).

In this SVM network, the training data was first collected from a single GC sensor, statistical features such as mean, standard deviation, and amplitude were then extracted to consolidate the data and improve training efficiency. Subsequently, the features vector was transformed from its original space into a higher‐dimensional space by selected kernel functions (linear, polynomial, radial basis, and sigmoid). Hyperplanes in the high‐dimensional space that can distinctly separate the data points and maximize the margin distance between classes were found. These hyperplanes act as decision boundaries for classifying newly generated data without any human assistance.

Six common motions (sitting down, climbing stairs, squatting, running, walking, and jumping) were considered in this study. Raw resistance data were directly collected by the GC sensor which was attached to a participant's knee. For each type of motion, the participant was required to move continuously for at least 60 s in order to collect multiple full motion cycles. The details of the collected data are visualized in Figure 4c. Based on the data distribution, 150 data points of each motion (within ≈12 s) were treated as one sample. After removing redundant data that appeared at the start and end of the motions, 118 samples were obtained in total, and individual sample sizes were represented in Table S3 (Supporting Information). These samples were split into a training set and a testing set with a ratio of 8:2. The training set was used to fit the model and the testing set was used to provide an unbiased evaluation of the trained model.

Moreover, mean, and standard deviation were selected to be the features for model training as they achieved the highest prediction accuracy among all combinations of statistical features.^[^ ^29^ ^]^ These features were extracted from the samples and then combined to form a feature matrix. Training the model based on the feature matrix achieved high classification accuracies of 95.83% with both linear and polynomial transformations. Their confusion maps are shown in Figure 4d,e. **Table** **1** compares the fabrication, sensing performance, and ML‐enabled human motion prediction accuracy between sensors reported in previous works and the GC sensor introduced in this study.

**Table 1:** The comparison of ML‐enabled human motion prediction.

Sensor	Preparation method	Sensing mechanism	ML algorithm	Motions	Precision	References
SWCNT‐PET/SP	Coating & Textile	Resistive	SVM	Walk, run, jump, and sprint	84	[32]
Textile‐Based TENG	Complex assembly	Voltage	Artificial neural network	Walk	–	[33]
Magnetic induction system	–	Magnetic	SVM	Knock, lift, throw, and walk	83.5	[34]
Caregiving walking stick	Complex assembly	Voltage	Deep learning	Ultra‐low frequency motion	100	[35]
Graphene/ethyl cellulose	Complex assembly	Current	First derivatives	Bend hand joint, grasp, stoop, and walk	<0.11*	[36]
MXenes ink electrodes	Complex assembly	Voltage	SVM	Walk, run, and jump	92.18	[29]
GC sensor	3D printing	Resistive	SVM	Walk, run, jump, sit, squat, and climb stair	95.83	This work

The sign “‐” denotes no related date.

The “*” denotes the Pearson correlation coefficient value.

### 2.5 Humidity Sensing Performance of the GC Sensor

**Figure 5:**
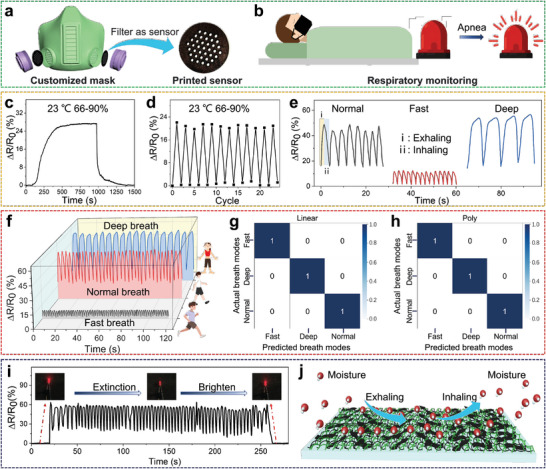
Humidity sensitivity and potential applications of the GC sensor. a) A filter with sensor function and b) its application scenarios; c) The resistance changes of the GC sensor from the indoor environment to the high humidity environment and back to the indoor environment; d) Cyclic testing of the GC humidity sensor; e) Respiration testing results of normal breath, fast breath, and deep breath; f) The output data obtained from different breath modes; Confusion maps for SVM outcome with g) linear h) and polynomial kernel functions; i) The signal detected by the GC sensor attached to the mask and the brightness change of the connected lamp during the test; j) Schematic illustration of moisture attachment and department from a GC sensor.

Humidity refers to the quantity of water vapor present in the air. By utilizing MJF printing technology, we produced a mask with an integrated sensor filter that can detect variations in breathing patterns by detecting changes in humidity levels caused by respiration (**Figure** **5**a). The integrated sensor consists of alternating layers of TPU and GC, which have the same structure as the strain sensor but with a distinct filter‐like shape. Its Δ*R*/*R*_0_ value changes with external humidity, resulting in a multifunctional material printed using MJF technology. Changes in the signal affected by humidity can help assess an individual's vital signs and determine the need for immediate medical intervention when respiration stops (Figure 5b).

Figure 5c shows the resistance change of the sensor from the indoor environment (humidity 66%) to the high‐humidity environment (humidity 90%) and back to the indoor environment. As the humidity level rises, the resistance change of the sensor reaches a plateau, indicating a balance between the humidity level of the sensor and that of the surrounding environment. When the sensor was taken out of the high‐humidity environment, the resistance declined swiftly and returned to its original state promptly. This indicates that the sensor is remarkably sensitive to humidity. The sensitivity of the GC sensor can be calculated as 0.87 from the curve of Δ*R*/*R*_0_ versus relative humidity (RH) ranging from 10% to 90% (Figure S9, Supporting Information). This sensitivity is reliable and can be sustained over multiple cycles, as shown in Figure 5d. Even after more than ten cycles, the variation in Δ*R*/*R*_0_ remains highly stable. Although changes in the humidity of the surrounding environment may slightly impact the initial resistance, the stability of the output signal remains unaffected. This suggests that the printed GC/TPU composite can be employed as a humidity sensor for monitoring environmental moisture.

In addition, the sensor was tested for three modes of breathing: normal, fast, and deep breathing. The characteristics of the output signal of the sensor can indicate the respiration rate and intensity for each mode (as shown in Figure 5e). This output signal provides an accurate representation of the respiration rates and modes, which can be correlated to various activities, including sleep, walking, and running. The statistical features of data collected from the GC humidity sensor were used to train an SVM for predicting the breathing modes of specific individuals (Figure 5f), including firefighters, patients, and individuals with disabilities. A total of 84 samples were obtained by considering 50 data points as one sample. These samples were linearly separable, making it easier for the SVM algorithm to identify clear decision boundaries. Therefore, the trained model achieved high classification accuracies of 100% with both linear and polynomial transformations. Their confusion maps are shown in Figure 5g,h. The findings indicate that a GC/TPU composite fabricated using MJF printing technology can serve as a reliable humidity sensor for precise prediction of breathing modes, with the aid of machine learning.

To verify the practicality of this humidity sensitivity, samples were printed inside the masks and tested. As shown in Figure 5i, as respiration progressed, the electric resistance of the sample increased, causing the connected diode to gradually darken and extinguish. When the mask was removed, the diode gradually brightened again. This demonstrates that the printed sample can serve as a reliable respiration monitor.^[^ ^37^ ^]^ Monitoring vital signs in certain scenarios, such as during fires or in environments with thick smoke and extreme heat, can be challenging. The MJF‐printed humidity sensor can provide a more effective means of monitoring human breath, enabling timely rescue efforts. The possible mechanisms are illustrated in Figure 5j, that is, the PVP, acidified CNTs, and TPU in the GC sensor absorb moisture in the environment, thereby decreasing the electrical resistance of the sensor. When the humidity decreases, the absorbed water is released into air due to the joule heat and humidity homeostasis, causing a sharp decrease in the value of Δ*R*/*R*_0_. Therefore, this approach holds practical implications in various fields, including but not limited to fire rescue, medical care, and environmental monitoring.

As shown in **Table** **2**, the 3D printing structure not only provides the sensor with good conformality to the human body but also enables it to monitor human motions and breaths. The sensitivity, detection range, and long‐term stability of the printed sensors are comparable, or even superior, to what has been reported in recent years. These results collectively affirm the excellent performance of the strain/humidity sensors created in this study. Furthermore, the multilayer sensors, printed with conductive and water‐affinity materials, hold the potential to function as a single sensing unit, detecting both strain and humidity simultaneously through electric resistance changes driven by structural deformation and moisture adsorption/desorption. A possible way to decouple the strain and humidity signal responses is by detecting the signals in different directions (e.g., longitudinal and transvers directions) of the sensor and training ML models for decoupling and prediction.^[^ ^38^ ^]^

**Table 2:** Comparison of performance of polymeric stain/humidity sensors reported in recent years.

Polymer	filler	Solvent	Fabrication method	Template	Application scenario	Sensitivity		Detection range		Stability		References
						S^a^	H^b^	S (%)	H (%)	S^c^	H^c^	
Liquid crystals	–	Chloroform	Spin coating	Yes	–	–	–	0–30	–	30	–	[4a]
Nylon fabrics	Carbon particle	Water	Dip‐coating	No	Human–machine interaction	1.5	6.27	0–30	30–80	5k	6	[4d]
Fax fabric	CNT	Acetone	Dip‐coating	No	–	1.24	4.73	5–20	10–90	20	30	[4b]
PDMS	Carbon materials	Water	Vacuum filtration	Yes	Motion monitoring	–	7	0–100	10–80	10k	10	[39]
PDMS	PAM	Water	Spin coating	Yes	Breathing state	–	20	0–25	22–98	–	4	[40]
Polyester yarn	MXene	Water	Dip‐coating	No	–	0.67	–	3–120	30–100	10k	–	[41]
PU	RGO	DMF	Spin coating	Yes	Monitoring humidity	–	0.12	0–60	10–70	10k	5	[42]
PVA	Ag nanoparticle	Water	Casting	Yes	Detect motion and humidity	1.6	–	1–200	30–90	200	5	[4c]
Organic hydrogel	–	Water	Casting	Yes	Humidity sensing	–	0.24	–	4–90	–	9	[43]
Polyacryl‐ amide	PEDOT:PSS	Water	Polymerization	Yes	Respiration and finger movement	0.31	0.44	100–700	23–85	1.1k	6	[44]
Agar	Polyelectrolyte	EG	Polymerization	Yes	Humidity sensing	–	–	–	29–76	3	3	[45]
PDMS	Sensor arrays	–	3D printing	Yes	–	18	0.07	0.7–1.5	20–80	4	–	[46]
Elastomer	CNTs	Xylene	3D printing	No	Deformation detection	8.94	0.04	0–60	55–90	10k	–	[47]
PA12	GC ink	Water	3D printing	No	Monitoring motions and breathing in a fire	20.1	0.87	0–70	10–90	1000	60	This work

The sign “‐” denotes no related date. “S” and “H” denote the strain sensor and humidity sensor, respectively. The sensitivity of the strain sensor (denoted as “a”) and humidity sensor (denoted as “b”) is demonstrated by the value of GF and the data of ΔR/R0%/%RH, respectively. The long‐term stability “c” denotes the maximum number of testing cycles reported in the literature.

## 3 Conclusion

In this study, we developed a novel GC ink and utilized MJF printing technology to fabricate a series of strain and humidity sensors that are both practical and scalable. These sensors exhibit exceptional conformity to both human joints and good mechanical performance such as flexibility and robustness. The output data obtained from the bending sensor are highly sensitive to the direction of deformation, and approving the sensor is well‐suited for monitoring human motions. The multilayered bending sensor has the capability to detect wrist flexion and recognize five basic emergency hand signals, including “go”, “stop”, “turn right”, “turn left”, and “in danger.” Furthermore, the output signals obtained from a bending sensor worn on the knee were used to train an SVM with 95.83% accuracy to predict six common motions, including walking, running, sitting down, squatting, jumping, and climbing stairs. The integration of machine learning and bending sensors holds great potential for predicting human motions and enabling timely rescue measures. Additionally, the excellent humidity sensitivity of the sensor makes it an excellent candidate for monitoring human breath. The change in electrical resistance can be reflected by the brightness of a lamp, which directly indicates vital signs. The improved thermostability and flame retardancy of the as‐fabricated GC sensors ensure their safety applications in fire rescuing. The utilization of advanced GC inks and fast MJF fabrication techniques has the potential to confer flexibility, robustness, and shape memory to strain‐humidity sensors, leading to significant benefits in human safety and healthcare.

## 4 Experimental Section

### Materials

GNPs (TNGNP, 2–16 µm, volume resistivity <0.15 Ω cm) and acidified CNTs were purchased from Chengdu Organic Chemicals Co. Ltd. Polyvinylpyrrolidone (Mw = 360 K) was purchased from Sigma–Aldrich Pte. Ltd. Ultrasint TPU01 powder was provided by HP Inc., USA.

### Preparation of the GC ink

CNTs and GNPs were ultrasonically dispersed in 50 mL water with 2 wt.% PVP, respectively. After ultrasonication for 3 h, the unexfoliated conductive fillers were removed by centrifuging at 3000 r min^−1^ for 30 min. The solid content of CNTs and GNPs in the solution was calculated by drying different volumes of the solution. The GC ink was obtained after mixing the two solutions together. The weight ratio of CNTs and GNPs in the ink was 1:1.

### Advanced Printing Process of MJF Testbed

The printing was performed by repeating the process of powder recoating, FA deposition, infrared irradiation, GC ink deposition, and powder coalescence. Each powder layer with a thickness of 60 µm was spread on the print bed at a recoating speed of 200 mm s^−1^. The supply ratio, which was the ratio of the ascending height of the supply bed to the descending height of the print bed, was set at 2 to ensure sufficient powder could be spread on the print bed. The FA dispensed from the printhead was selectively deposited onto the powder layer. The region with the FA was heated to 140–150 °C by infrared irradiation, and the powder particles were fused together after heat absorption. In contrast, the boundary region with the detailing agent was at a temperature lower than the melting point of TPU to ensure the dimensional accuracy of the printed specimens. Subsequently, the GC ink was selectively applied to the TPU layer and allowed to dry to create a conductive GC layer. A new layer of TPU powder was then spread on the print bed by the roller, and the process was repeated until the designed 3D specimens were printed. The printed specimens were cooled to room temperature and then cleaned by bead blasting.

The types and distribution of inks for MJF printing were adjustable, hence obtaining integrated sensors in a single printing process. The wearable sensors with different sizes were designed and printed based on the joint size of the volunteer (31 years old, 70 kg, 175 cm). Informed consent was obtained from the volunteers for recording of the body condition. The length and width of the strips and sensors were adjusted to well match the volunteer. Therefore, customized sensors could be fabricated in a rapid way. The GC sensors and the copper tapes were bonded together by silver adhesive to collect signals generated by the change of the sensor resistance when the human joints move. The two ends of the filter shape sensor were also taped with copper tape and placed in a printed mask to collect the resistance changes of the sensors during breathing.

### Characterization of the GC Ink

The morphology GC was characterized using a scanning electron microscope (SEM) (JSM‐5600 LV, JEOL, Japan). The morphology of CNTs was observed by transmission electron microscopy (TEM) (JEM‐2100F, Japan). The apparent viscosity of the GC ink was measured at a shear rate ramp from 0.1 to 100 s^−1^ by a rotational rheometer (DHR‐2, TA Instruments, USA) with a parallel plate geometry of 40 mm in diameter. The temperature distribution of the printed patterns was recorded by an infrared camera (A655sc, FLIR systems Inc., USA).

### Characterization of the GC Sensor

The morphology and fractured surfaces of the printed GC sensor were characterized using SEM and optical microscopy (SZX16, Olympus Corp., Japan). The four‐probe conductivity meter (HPS2662, HELPASS, China) with a measuring range from 0 to 2.0 MΩ cm was used to test the conductivity of printed samples. The conductivity of printed parts was tested by a source meter (Keithley 2450, Tektronix Inc., USA) with a source current of 1 µA. The mechanical properties of the printed parts were performed on the Shimadzu AGX 10 kN universal tester at a crosshead speed of 5 mm min^−1^. Five specimens were measured for each parameter set. Thermogravimetric analysis (TGA) was conducted using samples of ≈10 mg (obtained by cutting from the MJF‐printed specimens), which were heated from 50 to 800 °C at 20 °C min^−1^ under nitrogen and air conditions. Microscale combustion calorimetry was used to investigate the flammability characteristics of UPR composites according to ASTM D7309‐07. Samples of ≈5 mg were heated in a nitrogen atmosphere at a constant heating rate of 1 °C s^−1^ from room temperature to 650 °C. The decomposition products were mixed with oxygen (20 mL mi^−1^n) and then combusted in the combustion furnace (900 °C).

## Conflict of Interest

The authors declare no conflict of interest.

## Supporting information

Supporting Information

## Data Availability

The data that support the findings of this study are available in the supplementary material of this article.
