# Supplementary material for: 3D Printed Conformal Strain and Humidity Sensors for Human Motion Prediction and Health Monitoring via Machine Learning
Source: Adv Sci (Weinh). 2023 Nov 8;10(36):2304132. doi: 10.1002/advs.202304132 (PMC10754119; doi:10.1002/advs.202304132)
Supplement: Supplementary file 1 — Supporting Information [file ADVS-10-2304132-s001.pdf]

ADVANCED SCIENCE
Open Access

Supporting Information

for *Adv. Sci.*, DOI 10.1002/advs.202304132

3D Printed Conformal Strain and Humidity Sensors for Human Motion Prediction and Health Monitoring via Machine Learning

*Yanbei Hou, Ming Gao, Jingwen Gao, Lihua Zhao, Edwin Hang Tong Teo, Dong Wang, H. Jerry Qi and Kun Zhou**

3D Printed Conformal Strain and Humidity Sensors for Human Motion Prediction and Health Monitoring via Machine Learning

*Yanbei Hou, Ming Gao, Jingwen Gao, Lihua Zhao, Teo Hang Tong Edwin, Dong Wang, H. Jerry Qi, and Kun Zhou**

Y. Hou, M. Gao, L. Zhao, K. Zhou
HP-NTU Digital Manufacturing Corporate Lab
School of Mechanical and Aerospace Engineering
Nanyang Technological University
Singapore 639798, Singapore
Email: kzhou@ntu.edu.sg

Y. Hou, M. Gao, J. Gao, K. Zhou
Singapore Centre for 3D Printing
School of Mechanical and Aerospace Engineering
Nanyang Technological University
Singapore 639798, Singapore

L. Zhao
3D Lab, HP Labs
HP Inc.
Palo Alto, CA 94304, USA

T. Edwin
School of Electrical & Electronic Engineering
Nanyang Technological University
Singapore 639798, Singapore

D. Wang
School of Mechanical Engineering
Shanghai Jiao Tong University
Shanghai 200240, China

H. J. Qi
The George Woodruff School of Mechanical Engineering
Georgia Institute of Technology
Atlanta, GA 30332, USA

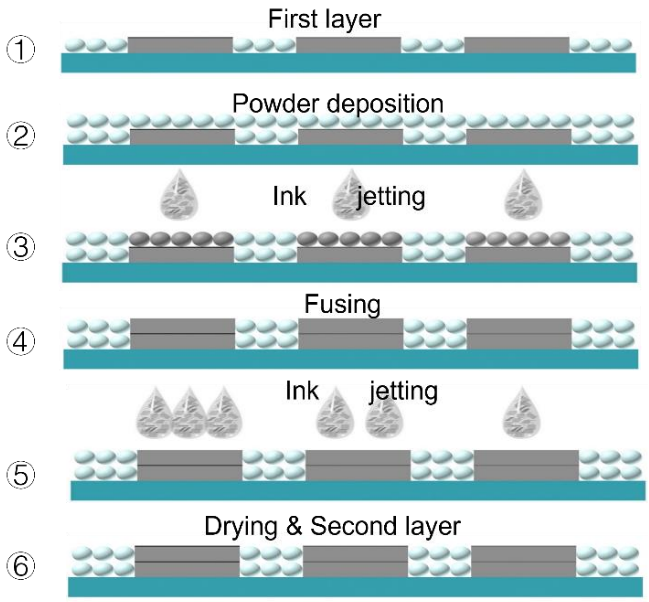

Figure S1. Advanced MJF printing process. A repeated unit is composed of fused TPU powder and dried GC ink. Following the deposition of TPU powder on the initial unit, the next step involved selectively spraying FA in accordance with the design. Upon heat absorption by the FA, the TPU powder particles fuse together to form a fresh TPU layer. Subsequently, the GC ink is selectively applied to the TPU layer and allowed to dry to create a conductive layer. These steps ultimately lead to the creation of the second unit. Units are stacked repeatedly to form a multilayered printing product.

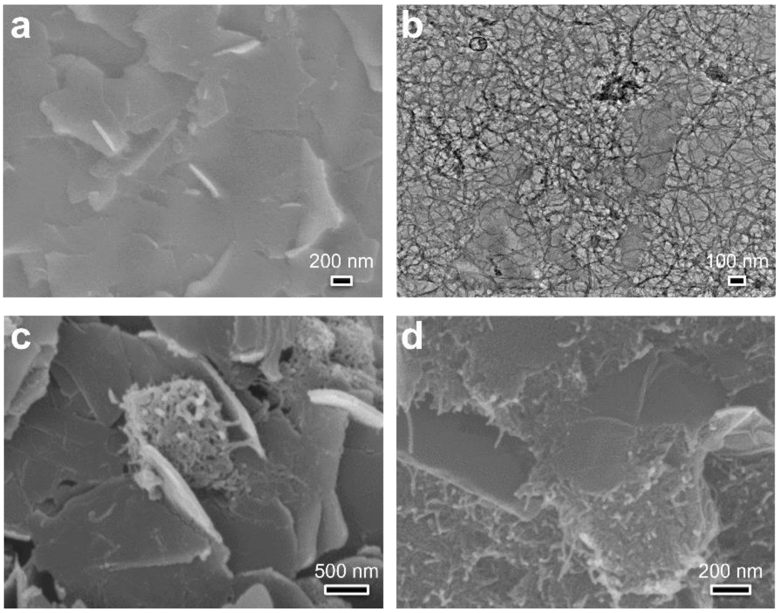

Figure S2. Characterization of GC ink. (a) SEM image of exfoliated GNPs; (b) TEM image of CNTs; (c) SEM image of the GC ink; (d) SEM fractography of the self-supporting GC film.

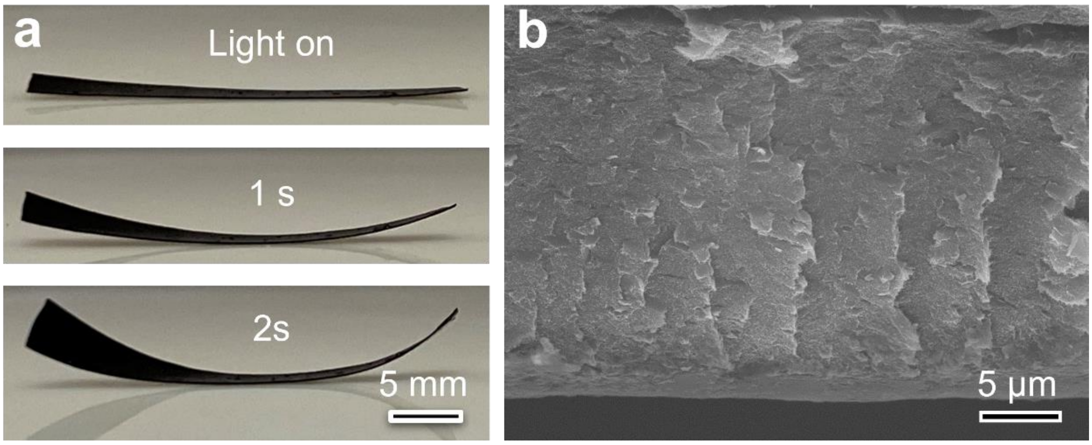

Figure S3. a The thermosensitive properties of the GC film. A lamp emitting radiation equivalent to 1 solar intensity was employed to thermally stimulate the GC film, which underwent gradual torsional deformation until it reached a stabilized conformation; **b** The fractured section of GC film.

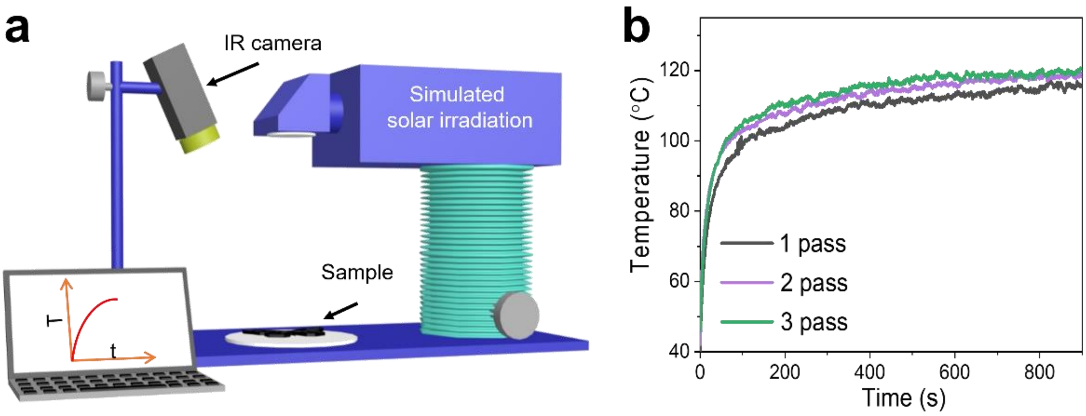

Figure S4. a Schematic illustration of the simulated solar irradiation setup; **b** The photothermal performance of GC ink deposited with different passes.

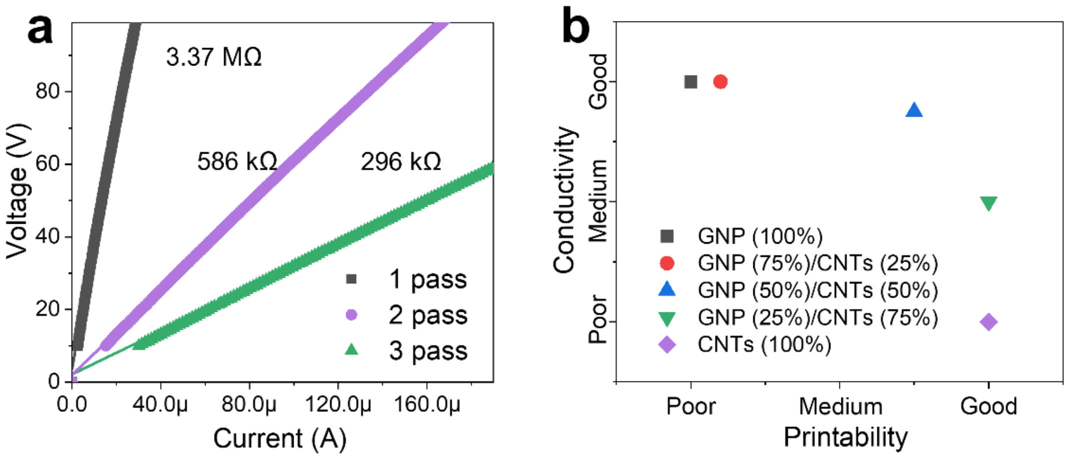

Figure S5. a The conductivity of printed parts with different passes; **b** The comparison

of conductivity and printability of GNP, CNTs, and GC inks. The electrical resistances of ink compositions that are not suitable for printing, namely GNP and GNP (75%)/CNTs (25%), were measured by analyzing films produced through ink drying. In contrast, the conductivities of other ink formulations were determined by examining printed samples created through a MJF testbed that utilized 3-pass ink jetting. The samples used for the conductivity test are of identical size to the GC/TPU samples that were printed.

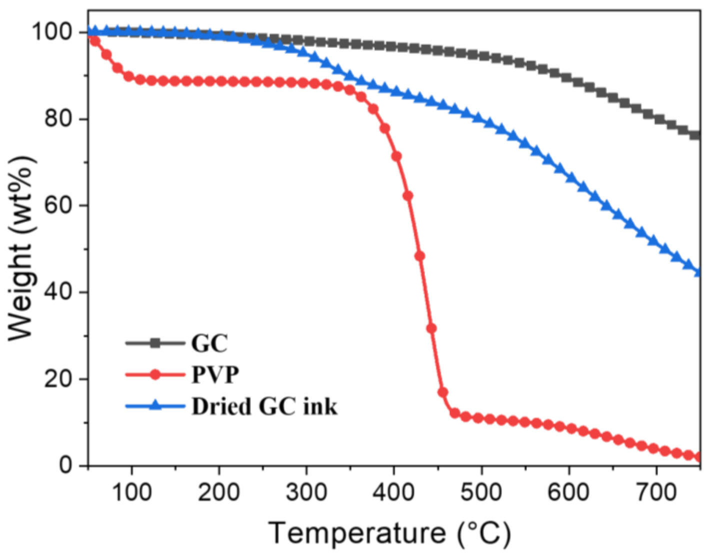

Figure S6. TGA results of GC hybrids, PVP, and dried GC ink. The final residue weights of GC, PVP, and dried GC ink were 75.70 wt%, 2.06 wt%, and 44.51 wt%, respectively. Based on the equation $M_{\mathrm{GC}}w + M_{\mathrm{PVP}}(1-w) = M_{\mathrm{dried\ GC}}$, where w is the weight percentage of GC in the dried GC ink, and M_{GC}, M_{PVP}, and $M_{\mathrm{dried\ GC}}$ are the final residue weight of GC, PVP, and dried GC ink, respectively. The content of GC in the ink was calculated to be 42.998%, which matched well with the designed composition.

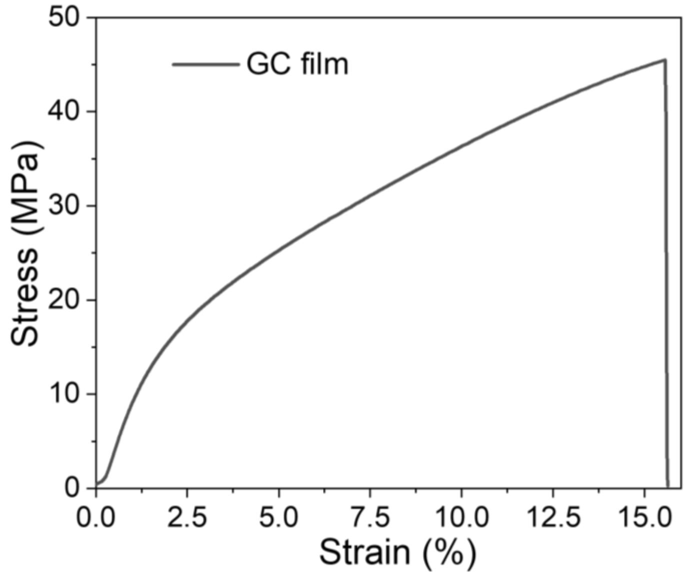

Figure S7. Strain-stress curve of GC film.

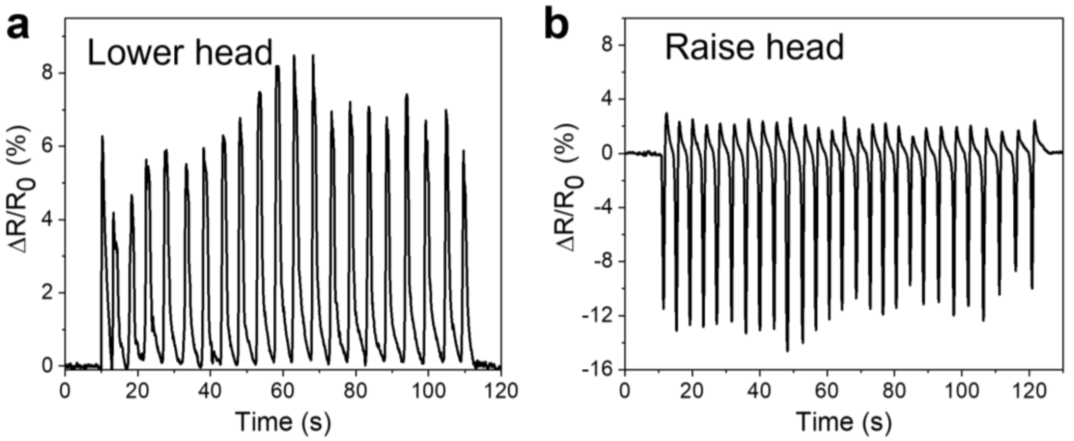

Figure S8. Output signals of the GC sensor collected at the bending of the neck. **a** lower head; **b** Raise head.

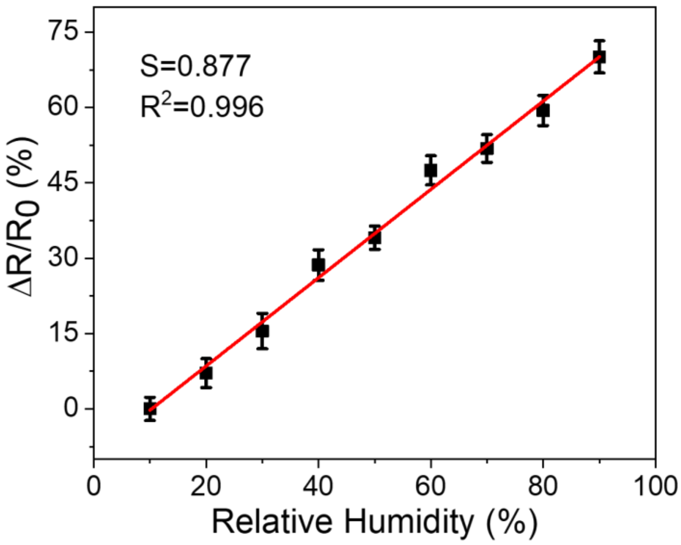

Figure S9. Relative resistance variation of GC sensor versus relative humidity ranging from 10% to 90%.

Table S1. The comparison of manufacturing approaches of strain/humidity sensors.

**Method**	**Advantage**	**Limitation**	**Representative work**
Spin coating	Simple; inexpensive	Lack of material efficiency; not suitable for large substrates	[1]
Dip coating	Simple; reliable	Unbalanced coverage; coating buildup	[2]
Casting	Low costs; great design flexibility; minimal setup time	Costlier materials; post-casting machining requirements	[3]

Polymerization	Fast; reliable	Expensive; strict reaction environment	[4]
Direct ink writing	Low consumption; multi-materials printing	Supporting substrates needed; not suitable for large parts	[5]
MJF	Fast printing speed; no need for supporting substrates; selective ink jetting; integration of conformal parts	Slightly rough surface, only single-color models	This work

Table S2. Data summary presented in the manuscript.

Sample	Conductivity (S/m)	Mechanical performance		Flame retardancy	
		Stress (MPa)	Elongation (%)	PHRR (W/g)	Ignition time (s)
GC film	3.18 ± 0.42	44.82 ± 1.24	15.17 ±2.27	-	-
TPU	-	8.36 ± 0.63	246.60 ± 8.86	396.2 ± 13.2	1.2 ± 0.1
1Pass/TPU	$(1.23 \pm 0.11)\times 10^{-5}$	10.46 ± 1.72	288.89 ± 7.57	335.5 ± 9.8	2.0 ± 0.2
2Pass/TPU	$(4.17 \pm 0.21) \times 10^{-3}$	12.49 ± 1.18	303.80 ± 11.32	309.6 ± 10.1	4.1 ± 0.2
3Pass/TPU	$(1.48 \pm 0.16) \times 10^{-2}$	10.15 ± 0.79	250.03 ± 10.92	287.1 ± 10.7	5.7 ± 0.3

The sign “-” denotes that the item was not tested in this work.

Table S3. Sample size distribution

Classes	Sample size
Climb stair	9
Jump	23
Squat up downs	38
Run	4
Walk	16
Sit down-stand up	28
Total	118
